# Diagnostic Aspects and Retinal Imaging in Ocular Toxocariasis: A Case Report from Italy

**DOI:** 10.1155/2012/984512

**Published:** 2012-04-03

**Authors:** Onelia Verallo, Serena Fragiotta, Francesca Verboschi, Enzo Maria Vingolo

**Affiliations:** Department of Ophthalmology, “S. M. Goretti” Hospital, University of Rome “La Sapienza”, Via G. Reni, 04100 Latina, Italy

## Abstract

*Toxocara canis* is a nematode parasite, commonly found in dogs. This roundworm parasite can invade the eye, causing visual impairment. *Toxocara* should be considered as a possible causative agent of posterior and diffuse uveitis, and it could be considered in the differential diagnosis of retinoblastoma. Ocular manifestations vary from severe endophthalmitis to silent incidental findings on a routine examination. We report a case of ocular toxocariasis in a 24-year-old Asiatic female that presented to us complaining of visual impairment. Fundoscopic examination revealed a posterior pole granuloma and exudative retinal detachment along with exudates. Presentation, clinical findings, morphological changes, and treatment are discussed. The enzyme-linked immunosorbent assay serology for *Toxocara canis* was performed, demonstrating the positivity for IgG and IgE. Treatment with the antihelminthic albendazole was initiated. Fluorescein angiography (FA; HRA 2, Heidelberg engineering) and optical coherence tomography (OCT; Spectralis, Heidelberg tomography) were performed, and results have been reported.

## 1. Introduction

Toxocariasis is the clinical term applied to infection in the human host with either *Toxocara canis *or *Toxocara cati *[1]. They are both ascarid nematodes, and humans can be infected by ingestion of soil or contaminated meat containing *Toxocara larvae*. Human seroprevalence for *Toxocara* antibodies varies with factors such as geographic location, socioeconomic status, and dietary habits [2]. The disease is unilateral in most cases, with mild to moderate intermediate or diffuse inflammation. Ocular toxocariasis usually manifests with a solitary chorioretinal granuloma, in the peripheral retina or in the macula, uveitis, and/or tractional retinal detachment. The most common clinical signs are vitreous inflammation, cystoid macular edema, and vitreoretinal traction strands leading to the optic disk and/or a granuloma [3]. We report a case of posterior pole granuloma and exudative retinal detachment as manifestation of ocular toxocariasis. The presentation, clinical findings, diagnosis, and treatment are discussed.

## 2. Case Presentation

**Figure 1:**
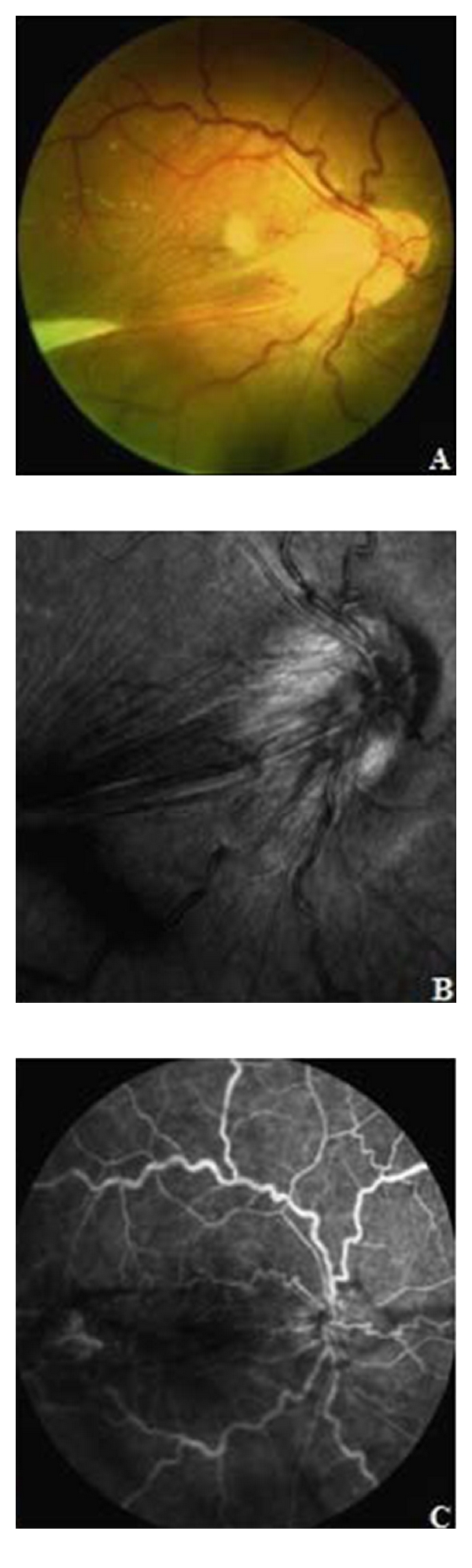
(A) Retinography shows a whitish mass at temporal side with vitreous bands between the mass and the optic disk, involving also macular region. (B) Infrared image obtained with FA, HRA 2, Heidelberg engineering, demonstrates an important traction of the optic disk. (C) Fluorescein angiography obtained with FA, HRA 2, Heidelberg engineering, shows leakage from the lesion.

A 24-year-old Asiatic female presented to our Department of Ophthalmology at the University of Rome “La Sapienza” in “S. M. Goretti” Hospital, complaining 15 days of decreased vision in the right eye. There was no previous ophthalmic history, and her general health was good. The patient denied having eye pain, redness, photophobia, or irritation. She was not taking any medications and denied any medications allergies. On presentation, the best corrected visual acuity (BCVA) in the affected eye was counting fingers (CFs) and 20/20 in the left eye, which were not correctable. Results of slit-lamp examination were normal. The bulbar conjunctiva was white and transparent, anterior chamber clear and cornea transparent. Furthermore, pupils were normal in size or shape and reactive to light, and no lens opacification was seen. Examination of the fundus revealed exudative retinal detachment with a large exudate located in the inferior temporal quadrant. A dense, white and well-circumscribed mass was located at 9 o'clock position. Furthermore, the vitreous exhibited opacities and sporadic cells. No abnormalities were seen in the left eye. Then the patient was hospitalized and we required further laboratory tests, which included blood count, erythrocyte sedimentation rate, leukocytes, liver function test, antinuclear antibodies, rheumatoid factor, purified protein derivative of tuberculin (with anergy panel), Toxoplasma IgG and IgM and *Toxocara* enzyme-linked immunosorbent assay (ELISA) with *Toxocara* excretory-secretory antigen (TES-Ag). Moreover, there was no evidence for an active infection with cytomegalovirus (CMV), HSV, VZV and Rubella. Radiological chest examination was normal. The results of laboratory tests showed eosinophils high respect to normal values, 18.2% (n.v. 0–5%) and absolute eosinophils count was 1.65 × 10^3^/mL (n.v.: 0–0.45 × 10^3^/mL). ELISA with TES-Ag was positive for IgG and IgE confirming the clinical appearance. The pharmacological treatment consisted of a 5-day course of oral albendazole 400 mg (10 mg/kg of body weight/day in two divided doses) two times a day. Six days after therapy, the patient presented cystoid macular edema (CME) treated with intravenous methylprednisolone. Funduscopic examination revealed vitreous strands with retinal traction and a whitish mass at temporal side with vitreous bands between the mass and the optic disk, involving also macular region (Figure 1(A)). Fluorescein angiography (FA; HRA 2, Heidelberg engineering) demonstrated a temporal lesion, possibly a granuloma with vitreous traction and diffuse ischemic area in the periphery. Lesion was characterized by central hyperfluorescence with early-phase dye leakage (Figures 1(B) and 1(C)).

**Figure 2:**
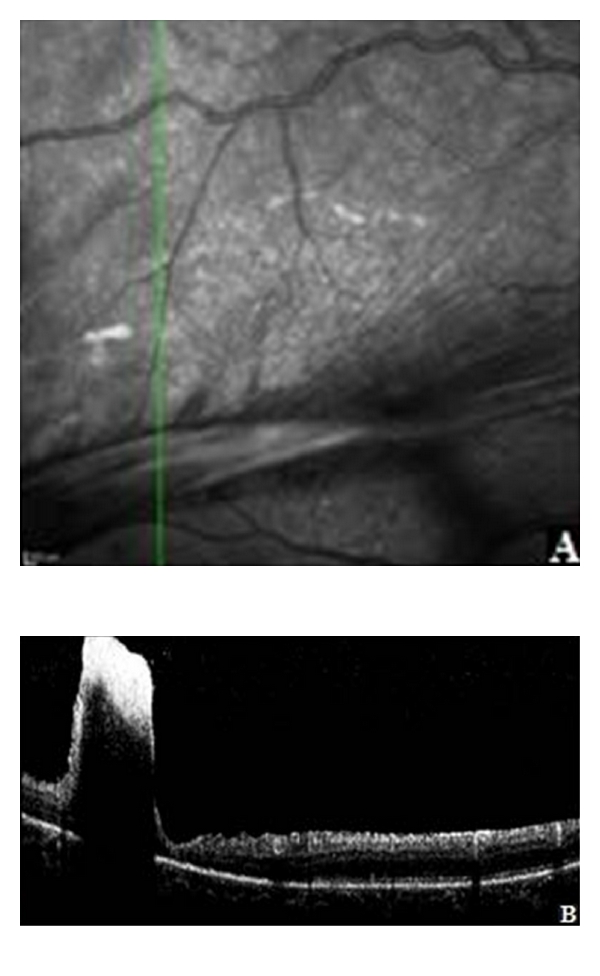
(A) Infrared image, obtained with OCT Spectralis, Heidelberg tomography, shows a prominent retinal fold in the right eye. (B) SD-OCT, vertical scan line of the lesion that shows a high reflectivity of the internal retinal layers with an area of low-absent reflectivity below which are not allowed the visualizations of the external retinal layers.

Optical coherence tomography (OCT; Spectralis, Heidelberg tomography) showed most important vitreoretinal tractions in macular region and optic disk, subretinal fluid, exudative macular lesion and central retinal thickness (CRT) was 524 *μ*m In the lesion side, the internal retinal layers presented a high reflectivity with an area of low-absent reflectivity below, which did not allow the visualization of the external retinal layers (Figures 2(A) and 2(B)).

The patient was proposed to surgery, consisting in vitrectomy to remove inflammatory tissues and relieve vitreomacular traction, but it was refused by the patient.

## 3. Discussion

*Toxocara canis* is a zoonotic disease transmission to human beings occurs by ingestion of embryonated eggs, usually from contaminated raw vegetables or infected raw meat (chicken, rabbit, and lamb) [4], contaminated water [1] or via geophagia. When *T. canis* eggs are ingested by human, the larvae penetrate the mucosal epithelium, and they remain developmentally arrested in the tissue phase (third-stage larvae). They show no growth or morphological differentiation and cannot complete their life cycle [5]. The larvae penetrate the bowel wall and migrate through vessels to muscles, liver, and lungs and sometimes to the eye and the brain. This prolonged migration is responsible for the chronic eosinophilia, while the widespread visceral invasion, particularly of liver and lung, results in minute multiple granulomata. The parasite accesses into the eye through blood circulation, via the ciliary vessels to the choroid or via the central retinal vessels to the retina and vitreous [6]. Common symptoms that bring patients to the clinic include blurred vision and floaters. Pain and photophobia may also be present, but they are typically mild. In young patients, the eye infection may not be noticed until they fail a school vision screening test or develop strabismus or leukocoria. Ocular toxocariasis may present clinically as a granuloma at the posterior pole in 25 to 50 percent of patients [3]. The reason of this predilection for the posterior pole is unclear although it has been suggested that nematodes prefer to lodge in small, perifoveal end arteries.

The primary causes of vision loss in patients with ocular toxocariasis depend largely on the localization and the severity of the inflammation. Vision is typically decreased in patients with *Toxocara *endophthalmitis due to inflammatory media opacities, cystoid macular edema and/or cataract formation. Posterior pole granulomas, in contrast, usually cause vision loss by direct involvement of the macula or optic disc, by secondary formation of retinal folds or epiretinal membranes or, rarely, by the development of choroidal neovascularization. The diagnosis of toxocariasis is presumptive because a definite diagnosis requires actual demonstration of the larva in the patient eye.

In this study we have reported a case of ocular toxocariasis characterized by posterior pole granuloma and exudative retinal detachment, and its subsequent development in scar, vitreous strands with retinal traction in macular region. In most of cases, it is difficult to establish the diagnosis of ocular toxocariasis based on clinical manifestations only, because ocular symptoms may be various and inflammatory signs are not always present. For these reasons, in the case presented here, we want to show diagnostic process and the morphological aspects observed with imaging methods as optical coherence tomography and fluorescein angiography. In this way, the clinical features along with instrumental tests can help the clinicians in the diagnostic process, even when low or undetectable *Toxocara *serum immunoglobulin titers are present. In our case, there was no signs of systemic involvement, but only the presence of eosinophilia at laboratory examinations and positivity of IgG and IgE. Higashide et al. [7] have shown a case of subretinal *Toxocara* granuloma evaluated with OCT and FA examinations. They concluded that granuloma may have a presentation similar to idiopathic choroidal neovascularization (CNV). In our opinion, this presentation occurs at the early stage of disease; indeed, several scientific studies [3, 6, 8] showed that the granuloma induces vitreous traction with some vitreous opacities, as also in our case. In conclusion, the diagnosis of *Toxocara canis* also requires the use of imaging methods to assess retinal changes, macular involvement and vitreous tractions or vitritis during followup and to evaluate therapeutic strategies.
